# Supplementary material for: Python-Microscope – a new open-source Python library for the control of microscopes
Source: J Cell Sci. 2021 Oct 6;134(19):jcs258955. doi: 10.1242/jcs.258955 (PMC8520730; doi:10.1242/jcs.258955)
Supplement: Reviewer comments [file joces-134-258955_review_history.pdf]

Python-Microscope - a new open-source Python library for the control of microscopes

David Miguel Susano Pinto, Mick A. Phillips, Nicholas Hall, Julio Mateos-Langerak, Danail Stoychev, Tiago Susano Pinto, Martin J. Booth, Ilan Davis and Ian M. Dobbie
DOI: 10.1242/jcs.258955

Editor: Jennifer Lippincott-Schwartz

Review timeline

Submission to Review Commons:	24 February 2021
Submission to Journal of Cell Science:	22 May 2021
Editorial decision:	12 July 2021
First revision received:	12 August 2021
Accepted:	23 August 2021

Reviewer 1

Evidence, reproducibility and clarity

Summary:
In this manuscript, Pinto et al. report Python-Microscope, a new open-source Python library for microscopy control. The new library lets microscope builders implement individual microscope devices as Python Classes with devices specific parameters and methods. Furthermore, the new Python library supports remote procedural calls and turns individual devices into a resource accessible over a network. Moreover, it has been designed to support hardware as well as software triggers. Finally, it provides several developer-friendly features; it is equipped with simple GUI programs for different device types, and it can simulate devices without the need for physical access to the hardware.

Major comments:
1. The authors highlight in their conclusion that the new Python library has the potential to accelerate and expand microscopy development. I agree with this statement since classes and methods do not need to be written in Python from scratch anymore. However, I would recommend that the authors include in their conclusion the value of the library for reproducibility if the final python acquisition code is shared along with publications. Nowadays, scientists frequently write in their publications that LabView or a specific commercial scope's acquisition software was used without any acquisition code. Python-Microscope would have the potential to change this trend, and the authors need to stress this aspect and its value for reproducibility in science accordingly.
2. The authors need to provide a more comprehensive overview of the currently used data acquisition strategies in their introduction. Currently, they highlight the acquisition software provided by vendors for data acquisition (mainly used by life scientists and not necessary scope builders/developers), Micro-Manager (mainly used by life scientists; currently also restricted to wide-field systems), and LabView (for advanced microscope systems; used by advanced developers). However, most advanced microscope builders use MatLab (Chmyrov et al. Nature Methods (2013) - https://doi.org/10.1038/nmeth.2556, Ta et al. Nature Communications (2015) - https://doi.org/10.1038/ncomms8977 , etc.), Python (York et al. Nature Methods (2013) - https://doi.org/10.1038/nmeth.2687, etc.), and LabView to write their acquisition software. Since the manuscript focused on advanced microscopes, the authors need to position their library with respect to Matlab and Python's current use as well.
3. The authors need to give (1) software provided by vendors, (2) LabView, and (2) Micro-Manager, more credit.

© 2021. Published by The Company of Biologists under the terms of the Creative Commons Attribution License (https://creativecommons.org/licenses/by/4.0/).

(1) Several microscope vendors (e.g., Abberior Instruments - https://imspectordocsseadthedocsio/en/latest/specpy.html) allow their scopes can be externally controlled to enable the execution of customer-driven acquisition strategies which the vendor's acquisition software itself might not have implemented with. The authors might want to include that scope vendors aim for more customer modifiable acquisition software.
(2) The authors criticize that LabView code can be hard to understand, reproduce and maintain. However, similar to writing good code in general, there are best practice strategies for writing good LabView code to ensure scalability, readability, and maintainability available as well (https://learn.ni.com/learning-paths/labview-core-3-2016-english). The primary problem might lie more on the side of lousy coding practice than on LabView's side to perform appropriately.
(3) The authors should include the current effort by Pinkard et al. (Pinkard et al. Nature Methods (2021) - https://doi.org/10.1038/s41592-021-01087-6) in their discussion.
(4) The authors might want to explain how they plan to facilitate the library's adoption and the long-term maintenance within the microscopy community. Do they plan to create a new category on Image.sc, which would allow the community to interact with the developers? etc. Furthermore, who will keep writing wrappers to the libraries provided by the vendors? etc Several useful software packages have been written in the past, but their existence was often not for long (after 2-3 years, most packages simply can not be used anymore). The concept of software maintenance is frequently not addressed/considered. Therefore, could the authors expand this aspect in an additional section of their paper?
(5) The authors stress using their library for complex scopes but do not provide an example of complex implementation (they only provide paper references). Only a code for a simple time-series is provided. It would be very beneficial to provide the code for implementing a complex microscope and its GUI with the author's library as separate figures or in the paper's supplement. This would also support point 1 in the review.

Minor comments:
1. It would help the paper if several phrases would be changed:
a. Title: 'Python-Microscope: High-performance control of arbitrarily complex and scalable bespoke microscopes." To: e.g., Python-Microscope: A new open-sources Python library for the control of microscopes Why? The authors use the word "high-performance" to address their Python library's trigger feature within the text. Unfortunately, that is not how most people would use the term for. Therefore, it should be avoided not only in the title but throughout the text. Furthermore, the word "complex" combined with microscopes should be avoided. A complex microscope is, for most microscope builders, a microscope that needs precise times and synchronization, includes several feedback active feedback loops, incorporates several devices, is very stable, etc. The context in which the term "complex microscopes" is used here is when the authors talk about the library's features to connect devices to servers either locally or remotely. I agree that the library can connect devices over arbitrary complex networks, but using the term "arbitrary complex microscopes" would be misleading considering the library's current speed limitations, the limited number of currently integrated devices, etc.
b. Various section titles:
"Libraray features" would be more suitable than "Use Cases" since the individual new features at the new library are described in this section. Also, the description of the individual features should be mentioned more accurately. The following list might be a better, more accurate fit: (1) "Device modularity" instead of "Device independence." Also, the current title "Write once, run with any device" is inaccurate since the wrapper for multiple devices has not been implemented. (2) "Experiment- and scope-specific layout" instead of "Experiments as programs." (3) "Complex network integration" instead of "Easy implementation of complex systems and scalability" (see reasoning under point a.) (4) "Hardware and software trigger integration" instead of "High performance, " (5) "Developer-friendly programming features" instead of "Simple development tool."
c. The authors should avoid using the term "Microscope" when talking about "Python-Microscope." It facilitates the manuscripts readability since it is occasionally not evident in the paper if they refer to the library or a microscope.
d. The authors should avoid the phrase "pythonic software platform" in the abstract since Python-Microscope is a library / Python package and not a software platform. Furthermore, the term "pythonic" describes the desired way to write Python code. It means code that does not just get the syntax right but follows the Python community conventions and uses the language in the way it is intended to be used. Instead, it might be advisable to write, "Python-Microscope offers elegant Python-based tools to control microscopes...".

© 2021. Published by The Company of Biologists under the terms of the Creative Commons Attribution License (https://creativecommons.org/licenses/by/4.0/).

2. Figure 1 should be supported by comments, e.g., #Load packages, #Parameter Initialization, #Create Devices, # Set camera parameters, etc.
3. The paragraph under the section "Experiments as programs" about the advantages of using Python (starting from "We have developed the software in Python, ...") should be moved into the Introduction section.

Significance

The field of microscopy emphasizes more and more openness and transparency of methods and tools being used to accelerate science, but also to guarantee reproducibility.

The authors' library is another step in the right direction. It is open, transparent, tries to satisfy multiple tool developers' needs to make the development of microscopes faster, easier, and more approachable/user-friendly. Although it can not yet be used for arbitrarily complex microscopes, it has the potential to do so in the future. For now, the authors need to manage to incorporate and involve microscopy developers' needs and requirements in the best possible way to be able to design the library as holistic as possible.

I am a physicist and microscope builder and have so far used MatLab, LabView, and Imspector as well as Python scripts to control microscopes, and I will definitely test the authors' library on my own.

Reviewer 2

Evidence, reproducibility and clarity

This manuscript describes Python-Microscope, a library/framework written in Python to control custom-built microscopes. Modern light microscopes consist of many computer controllable components and data sensors, and software has become an integral component of such systems. Microscopy is such a fast moving and diverse technology that a significant (>25%?) fraction of microscope systems can not be cookie-cutter, standardized systems, but are custom-built, assembled using commercial microscope stands and/or hardware from vendors such as Thorlabs. For many, creating the software to control such custom-built systems is more laborious and difficult than building the actual optical setup, and software toolkits to make this easier (such as the one presented in this manuscript) are of great interest to everyone working in this area. Python is at the moment probably the most widely used computer programming language by scientists, and a well-thought-out environment for microscope control from the Python language is a welcome addition.

The introduction does a good job describing the current situation (using multiple software from multiple vendors simultaneously, Micro-Manager, Labview), although it could be highlighted a bit more that several groups have created custom Python code for microscope control (such as https://github.com/ZhuangLab/storm-control, https://github.com/Ulm-IQO/qudi, https://github.com/fedebarabas/tormenta, https://github.com/AndrewGYork/tools), some with at least the hope that their code will be generally usable. It also could be noted that the Micro-Manager device abstraction layer has been accessible from Python for more than a decade (currently the Python 3 interface is at https://github.com/micromanager/pymmcore).

Manuscripts describing software tools have to balance the goal to "announce" and advertise the software package with the goal to objectively explain the design principles and choices made. In my opinion, this manuscript finds a nice balance, and leaves the reader with a decent understanding of the capabilities, advantages, limitations and high level architecture of the Python-Microscope package. Possible exceptions are the use of

© 2021. Published by The Company of Biologists under the terms of the Creative Commons Attribution License (https://creativecommons.org/licenses/by/4.0/).

the word "elegant" in the abstract, and extensive use of the word "bespoke" that I mainly know from real estate agent language and that likely is confusing to many readers for whom English is a second language.

As far as I am aware, "Microscope" is the most developed microscope abstraction layer written in pure Python. Remarkably, its design (device classes that inherit from a device-base class and have their own function calls, supplemented with "Settings" that can be declared by each device), is extremely similar to that of the Micro-Manager device abstraction layer (where "Settings" are called "Properties"), with the main difference being that one is written in Python and the other in C++ with C bindings. Writing these device classes in Python hopefully brings the advantage that more people can write them, however, the Micro-Manager C interface has the advantage that it can be used from any programming language on any platform, hence is more future proof than pure Python code. The downside of having multiple microscope device abstraction layers is that resources will be diluted and confuse partners in industry (which toolkit should they support with their limited resources?).
The number of devices supported is currently much, much greater in the Micro-Manager platform than in Microscope, and a translation layer to make Micro-Manager device adapters in Microscope does not seem out of the question, and could possibly benefit many.

Expected audience:
This manuscript will be of interest to those scientists who build/assemble their own microscope systems and write software code to control their operation.
Field of expertise:
I think a lot about microscope control software and how it can help scientists do their experiments.

Significance

see above.

Reviewer 3

Evidence, reproducibility and clarity

Pinto et al present a new python based software to control microscopes.
Overall the work is very interesting and will help microscopists to accelerate their development by providing new tool to integrate the different hardwares.

A few aspects commented below need to be clarified to help potential future users to integrate the software for the correct microscopes/hardware.

In general the software is mostly targeted to developers that want to build microscopes. as they mention in the discussion . Some positive features are (1) the ability to have experiments as scripts, (2) the software triggering,(3) the device-server structure, and (4) the ability to have virtual devices to try out the code and the testing I see in the github page. I think it's robust especially and mostly for the device-layer of the software. It's also positive that one can install it in python and import it in your programs, so it can be incorporated into other software fairly easy.

I miss more information regarding the latency of the device-server and software triggering, how fast can it be? How much delay would you have between

© 2021. Published by The Company of Biologists under the terms of the Creative Commons Attribution License (https://creativecommons.org/licenses/by/4.0/).

computers/devices? For example, could we have the devices sincronized at the microsecond range? I think this is super important so that the reader knows if it's worth using a software triggering approach with Python-Microscope or they should buy a DAQ instead.
It's good though that they don't want to limit themselves to software triggering but also mention hardware triggering,but it's important to better explain where are the limitations.

I also miss information about the triggering, do the software offer a platform that can synchronize devices, or that's more left to the developer to do? They say they can generalize to arbitrarily complex devices so therefore Ithinkit needs to be specified how. Same with the server feature, how fast is that link?

Some critical comments are that,first of all there are not so many drivers yet available (for example Hamamatsu camera). Iguess this paper is also to show proof of concept and then upon interest they will include more devices,but in that case it should be more documented how one can contribute to the project and generate new drivers . For example,if we want to try it tomorrow in our setups,and we have a specific device such as an Hamamatsu camera, What should we do? Should we contact the authors, write an issue in the github page or write the driver ourself?

Second.the graphical interface is maybe good enough for developers and builders but in order to have a solid microscope that biologists are going to useit needs a bit more work in that direction.

Significance

Microscope control software.especially is open source, can help the rapid integration of new hardware and accelerate overall microscopy development.

I see this paper as an important starting point platform for future more user friendly Python- microscope controlling software.

Author response to reviewers' comments

Reviewer #1 (Evidence, reproducibility and clarity (Required)):

Summary:

In this manuscript, Pinto et al. report Python-Microscope, a new open-source Python library for microscopy control. The new library lets microscope builders implement individual microscope devices as Python Classes with devices specific parameters and methods. Furthermore, the new Python library supports remote procedural calls and turns individual devices into a resource accessible over a network. Moreover, it has been designed to support hardware as well as software triggers. Finally, it provides several developer-friendly features; it is equipped with simple GUI programs for different device types, and it can simulate devices without the need for physical access to the hardware.

Major comments:

1. The authors highlight in their conclusion that the new Python library has the potential to accelerate and expand microscopy development. I agree with this statement since classes and methods do not need to be written in Python from scratch anymore.

© 2021. Published by The Company of Biologists under the terms of the Creative Commons Attribution License (https://creativecommons.org/licenses/by/4.0/).

However, I would recommend that the authors include in their conclusion the value of the library for reproducibility if the final python acquisition code is shared along with publications. Nowadays, scientists frequently write in their publications that LabView or a specific commercial scope's acquisition software was used without any acquisition code. Python-Microscope would have the potential to change this trend, and the authors need to stress this aspect and its value for reproducibility in science accordingly.

This is a good point. We have added the following to the discussion section.

"A further advantage of the approach provided by Microscope is in increasing reproducibility in science. Scientists frequently write in their publications that LabView or a specific commercial scope's acquisition software was used without any specific acquisition settings, code or macros to assist with reproduction. This is especially critical in complex experimental setups where specifics of acquisition are particularly important. Microscope has the potential to change this trend, allowing authors to freely publish simple code demonstrating exactly how their control and acquisition operates. Additionally, the defined device interfaces allow such code to be ported to other specific hardware with minimal changes."

2. The authors need to provide a more comprehensive overview of the currently used data acquisition strategies in their introduction. Currently, they highlight the acquisition software provided by vendors for data acquisition (mainly used by life scientists and not necessary scope builders/developers), Micro-Manager (mainly used by life scientists; currently also restricted to wide-field systems), and LabView (for advanced microscope systems; used by advanced developers).
However, most advanced microscope builders use MatLab (Chmyrov et al. Nature Methods (2013) - https://doi.org/10.1038/nmeth.2556, Ta et al. Nature Communications (2015) - https://doi.org/10.1038/ncomms8977 , etc.), Python (York et al. Nature Methods (2013) - https://doi.org/10.1038/nmeth.2687, etc.), and LabView to write their acquisition software. Since the manuscript focused on advanced microscopes, the authors need to position their library with respect to Matlab and Python's current use as well.

We thank the reviewer for pointing out the omission of Matlab control solutions and extending the references to other Python based approaches. We have also added a reference to the Pycro-Manager framework for Micro-Manager which has been published since our original submission.

We have added Matlab to the LabVIEW generalised control software section which now reads:

"custom control software often in LabVIEW or Matlab, both proprietary software. LabVIEW offers a visual programming environment that is commonly used for building instruments in the physical sciences, whereas Matlab is a programming platform with a focus on numeric computing."

And extended the description sections in the introduction with the following paragraphs and references:

"Matlab is a numerical focused programming environment that scientists are often familiar with for data processing. It has frequently been used for microscopy, leveraging a number of available Matlab sub packages to provide GUI's and easy access to complex data processing steps. The use of Matlab for microscope control is common in the field but the actual code is rarely shared and often custom to a single microscope setup and associated to image reconstruction (Chmyrov et al., 2013, Ta et al., 2015). Exceptions are ScanImage for the control of laser scanning microscopes (Pologruto et al., 2003), and Matlab Instrument Control (MIC) for the control of individual microscope components (Pallikkuth et al., 2018). Matlab provides a textual programming language simplifying code sharing and version control, however, Matlab is proprietary closed source software and the general requirement of many extensions significantly adds to the cost of implementing many systems."

"There is currently an increasing number of software options for microscope control in Python, many of which are in the form of custom scripts specific to a microscope (Alvelid and Testa, 2019, York et al., 2013) but some provide a fully integrated microscope control environments, namely

© 2021. Published by The Company of Biologists under the terms of the Creative Commons Attribution License (https://creativecommons.org/licenses/by/4.0/).

PYME https://www.python-microscopy.org/ for SMLM and ACQ4 (Campagnola et al., 2014) for electrophysiology. While this code is freely available and can be modified, their design around a specific setup, technique, or environment reduces its potential for code reuse in other projects.”

3. The authors need to give (a) software provided by vendors, (b) LabView, and (c) Micro-Manager, more credit.
(a) Several microscope vendors (e.g., Abberior Instruments - https://imspectordocs.readthedocs.io/en/latest/specpy.html) allow their scopes can be externally controlled to enable the execution of customer-driven acquisition strategies which the vendor's acquisition software itself might not have implemented with. The authors might want to include that scope vendors aim for more customer modifiable acquisition software.

The reviewer makes a good point, especially in the fact that a number of microscope vendors provide Python interfaces for their systems. We have added the following text: Several microscope vendors, such as Abberior Instruments and Zeiss, provide Python interfaces to enable instrument control from Python. These are all very useful additions to proprietary systems, however they have a fundamental draw back that each manufacture produces their own abstractions meaning code from one system is not compatible with another. Although these interfaces leverage the substantial Python infrastructure they are not generalisable and hence fail to enhance portability or reproducibility.

The fact that these companies are providing Python interfaces to their instruments indicates the general interest of the community in Python as a programming language to extend hardware capabilities. This demonstrates the potential benefit of an entirely Python based interface to a wide range of hardware.

(b) The authors criticize that LabView code can be hard to understand, reproduce and maintain. However, similar to writing good code in general, there are best practice strategies for writing good LabView code to ensure scalability, readability, and maintainability available as well (https://learn.ni.com/learning-paths/labview-core-3-2016-english). The primary problem might lie more on the side of lousy coding practice than on LabView's side to perform appropriately.

This is a fair point and we have revised the manuscript as indicated below. However, it remains true that it is much harder for a non-expert to write high quality code in LabView than in Python. This is particularly evident in complex systems.

We have changed the section about LabView to read:

“The visual nature of the programming environment makes simple projects easy but systems with a large number of hardware components or complicated control architecture can become hard to understand, reproduce, and maintain. Although this complication can be reduced with good programming practices, it is not uncommon to outsource such work to a commercial company \citep{chhetri2020software} because good code writing in LabView is significantly more challenging than in popular general purpose languages such as Python. Additionally, the LabView work flow does not integrate well into modern distributed source control infrastructure such as mercurial or git, a necessity for modern open source development.”

(c) The authors should include the current effort by Pinkard et al. (Pinkard et al. Nature Methods (2021) - https://doi.org/10.1038/s41592-021-01087-6) in their discussion.

A pre-print version of this paper was available on arXiv and cited in our original submission. Now this paper is published we have included the published reference and the following text has been added to our discussion section.

“As mentioned in the introduction, micromanager has a recently introduced Python interface, Pycro-Manager (Pinkard et al. 2021). This simplifies connections between micromanager based hardware interfaces and Python based analysis and control. Although this reduces the effort in

© 2021. Published by The Company of Biologists under the terms of the Creative Commons Attribution License (https://creativecommons.org/licenses/by/4.0/).

using Python for control and online analysis compared to other approaches it does not provide direct access to the hardware via Python. This interface keeps the existing micromanager infrastructure. Particularly new hardware interfaces still need code in both C/C++ and Java before they are accessible via the Python interface.”

4. The authors might want to explain how they plan to facilitate the library's adoption and the long-term maintenance within the microscopy community. Do they plan to create a new category on Image.sc, which would allow the community to interact with the developers? etc. Furthermore, who will keep writing wrappers to the libraries provided by the vendors? etc

This is a critical point, as the reviewer states, community involvement is essential to continuation of the project and provide a useful tool going forward. We have already published several systems utilising this software platform and are working hard to expand its user base.
We have asked for people to post question on the image.sc forums (https://image.sc/) and we also interact with developers and users on the github issue pages (https://github.com/python-microscope/microscope/issues). We have recently implemented a fully automated microscope on a simple motorized stand from Zaber. This provides a fully automated microscopy solution for a very low cost.

We have edited the end of the discussion to read

Microscope is a free and open source project currently being used in several labs with an open development approach. Our aim is that the microscope development community will find it a useful tool and engage in this development to increase its general usefulness. With that aim in mind, we perform our development conversations and user support in the open as github issues and the project is an image.sc community partner. In particular, expanding the number of devices supported by Microscope would be extremely beneficial. However, adding support for a device requires physical access to the device and the current list of supported devices echoes the devices we and our collaborators have access to. This is a chicken and egg problem. Python-Microscope needs broad device support to be widely adopted by the community but it needs contributions from the community to support those devices. We believe that, Microscope currently provides enough devices and infrastructure to support adoption by more developers. There are contribution guidelines within the ``Get Involved'' section of the documentation, available online at https://www.python-microscope.org/doc/get-involved.

5. The authors stress using their library for complex scopes but do not provide an example of complex implementation (they only provide paper references). Only a code for a simple time-series is provided. It would be very beneficial to provide the code for implementing a complex microscope and its GUI with the author's library as separate figures or in the paper's supplement. This would also support point 1 in the review.

The GUI elements provided by Python-Microscope are deliberately minimal implementations to allow basic connectivity and functionality of specific hardware to be tested. Python- Microscope is specifically designed to provide a hardware interface layer separate from the user interface. We provide a very simple examples to demonstrate how easily devices can be controlled. For more complete examples we have developed two associated packages providing GUIs, both are referenced in the text, BeamDelta is an optical alignment tool, while Microscope-Cockpit provides a full user interface to complex microscope systems. We have added a supplemental figure demonstrating the full GUI provided by Cockpit.

Minor comments:

6. It would help the paper if several phrases would be changed:
a. Title: 'Python-Microscope: High-performance control of arbitrarily complex and scalable bespoke microscopes." To: e.g., Python-Microscope: A new open-sources Python library for the control of microscopes

Why? The authors use the word "high-performance" to address their Python library's trigger feature within the text. Unfortunately, that is not how most people would use

© 2021. Published by The Company of Biologists under the terms of the Creative Commons Attribution License (https://creativecommons.org/licenses/by/4.0/).

the term for. Therefore, it should be avoided not only in the title but throughout the text.
Furthermore, the word "complex" combined with microscopes should be avoided. A complex microscope is, for most microscope builders, a microscope that needs precise times and synchronization, includes several feedback active feedback loops, incorporates several devices, is very stable, etc. The context in which the term "complex microscopes" is used here is when the authors talk about the library's features to connect devices to servers either locally or remotely. I agree that the library can connect devices over arbitrary complex networks, but using the term "arbitrary complex microscopes" would be misleading considering the library's current speed limitations, the limited number of currently integrated devices, etc.

We have changed the title to:
Python-Microscope: A new open-source library for the control of microscopes

b. Various section titles:
"Library features" would be more suitable than "Use Cases" since the individual new features at the new library are described in this section. Also, the description of the individual features should be mentioned more accurately. The following list might be a better, more accurate fit: (1) "Device modularity" instead of "Device independence." Also, the current title "Write once, run with any device" is inaccurate since the wrapper for multiple devices has not been implemented. (2) "Experiment- and scope-specific layout" instead of "Experiments as programs." (3) "Complex network integration" instead of "Easy implementation of complex systems and scalability" (see reasoning under point a). (4) "Hardware and software trigger integration" instead of "High performance, " (5) "Developer-friendly programming features" instead of "Simple development tool."

We have renamed the specified sections and subsections title and expanded the description in the list of use cases to be more accurate.

c. The authors should avoid using the term "Microscope" when talking about "Python-Microscope." It facilitates the manuscript's readability since it is occasionally not evident in the paper if they refer to the library or a microscope.

We have changed “Microscope” to “Python-Microscope” in multiple places of the manuscript where it was unclear whether we were referring to the software or to a physical microscope.

d. The authors should avoid the phrase "pythonic software platform" in the abstract since Python-Microscope is a library / Python package and not a software platform. Furthermore, the term "pythonic" describes the desired way to write Python code. It means code that does not just get the syntax right but follows the Python community conventions and uses the language in the way it is intended to be used. Instead, it might be advisable to write, "Python-Microscope offers elegant Python-based tools to control microscopes...".

We have changed the abstract as suggested.

7. Figure 1 should be supported by comments, e.g., #Load packages, #Parameter Initialization, #Create Devices, # Set camera parameters, etc.

Comments have been added the sample code.

8. The paragraph under the section "Experiments as programs" about the advantages of using Python (starting from "We have developed the software in Python, ...") should be moved into the Introduction section.

We have moved this segment to the end of the introduction.

Reviewer #1 (Significance (Required)):
The field of microscopy emphasizes more and more openness and transparency of methods and

© 2021. Published by The Company of Biologists under the terms of the Creative Commons Attribution License (https://creativecommons.org/licenses/by/4.0/).

tools being used to accelerate science, but also to guarantee reproducibility.

The authors' library is another step in the right direction. It is open, transparent, tries to satisfy multiple tool developers' needs to make the development of microscopes faster, easier, and more approachable/user-friendly. Although it can not yet be used for arbitrarily complex microscopes, it has the potential to do so in the future. For now, the authors need to manage to incorporate and involve microscopy developers' needs and requirements in the best possible way to be able to design the library as holistic as possible.

I am a physicist and microscope builder and have so far used MatLab, LabView, and Imspector as well as Python scripts to control microscopes, and I will definitely test the authors' library on my own.

Reviewer #2 (Evidence, reproducibility and clarity (Required)):

This manuscript describes Python-Microscope, a library/framework written in Python to control custom-built microscopes. Modern light microscopes consist of many computer controllable components and data sensors, and software has become an integral component of such systems. Microscopy is such a fast moving and diverse technology that a significant (>25%?) fraction of microscope systems can not be cookie-cutter, standardized systems, but are custom-built, assembled using commercial microscope stands and/or hardware from vendors such as Thorlabs. For many, creating the software to control such custom-built systems is more laborious and difficult than building the actual optical setup, and software toolkits to make this easier (such as the one presented in this manuscript) are of great interest to everyone working in this area. Python is at the moment probably the most widely used computer programming language by scientists, and a well-thought-out environment for microscope control from the Python language is a welcome addition.

1) The introduction does a good job describing the current situation (using multiple software from multiple vendors simultaneously, Micro-Manager, Labview), although it could be highlighted a bit more that several groups have created custom Python code for microscope control (such as https://github.com/ZhuangLab/storm-control, https://github.com/Ulm-IQO/qudi, https://github.com/fedebarabas/tormenta, https://github.com/AndrewGYork/tools), some with at least the hope that their code will be generally usable. It also could be noted that the Micro-Manager device abstraction layer has been accessible from Python for more than a decade (currently the Python 3 interface is at https://github.com/micro-manager/pymmcore).

We have significantly expanded the references to previous Python code and made other changes to the relevant sections as detailed in the response to reviewer #1 and quoted below. We have made reference to the recently published Pycro-Manager package (the previous version referenced the arXiv preprint of this paper. It should be noted that although the Python bindings for mmcore have been available for more than a decade, they have been rarely used, the only published paper referencing them appears to be the whitepaper from a workshop on microscope control software published on arXiv in 2020 (https://arxiv.org/abs/2005.00082).

“There is currently an increasing number of software options for microscope control in Python, many of which are in the form of custom scripts specific to a microscope (Alvelid and Testa, 2019, York et al., 2013) but some provide a fully integrated microscope control environments, namely PYME https://www.python-microscopy.org/ for SMLM and ACQ4 (Campagnola et al., 2014) for electrophysiology. While this code is freely available and can be modified, their design around a specific setup, technique, or environment reduces its potential for code reuse in other projects”

2) Manuscripts describing software tools have to balance the goal to "announce" and advertise the software package with the goal to objectively explain the design principles and choices made. In my opinion, this manuscript finds a nice balance, and leaves the reader with a decent understanding of the capabilities, advantages, limitations and high level architecture of the Python-Microscope package. Possible exceptions are the use of the word "elegant" in the abstract, and extensive use of the word "bespoke" that I mainly

© 2021. Published by The Company of Biologists under the terms of the Creative Commons Attribution License (https://creativecommons.org/licenses/by/4.0/).

> know from real estate agent language and that likely is confusing to many readers for whom English is a second language.

We have reworded the abstract to say

“Python-Microscope offers simple to use Python-based tools to control microscopes…”

We use the term “bespoke” to refer to the construction of novel optical microscopes, as opposed to controlling existing integrated systems from commercial vendors. We have reworded paper to refer to custom built microscopes and optical systems to clarify this point.

> As far as I am aware, "Microscope" is the most developed microscope abstraction layer written in pure Python. Remarkably, its design (device classes that inherit from a device-base class and have their own function calls, supplemented with "Settings" that can be declared by each device), is extremely similar to that of the Micro-Manager device abstraction layer (where "Settings" are called "Properties"), with the main difference being that one is written in Python and the other in C++ with C bindings. Writing these device classes in Python hopefully brings the advantage that more people can write them, however, the Micro-Manager C interface has the advantage that it can be used from any programming language on any platform, hence is more future proof than pure Python code. The downside of having multiple microscope device abstraction layers is that resources will be diluted and confuse partners in industry (which toolkit should they support with their limited resources?). The number of devices supported is currently much, much greater in the Micro-Manager platform than in Microscope, and a translation layer to make Micro-Manager device adapters in Microscope does not seem out of the question, and could possibly benefit many.

We are aware of the similarity between our approach and that in micromanager. There is therefore significant overlap and possible duplication of effort, however when we started this project we reviewed the Python bindings of micromanager core and felt that using this approach would add significantly, not only to our development effort, but also to end user effort as they would also have to install Micro Manager and its Python bindings. In addition, we believe that there is significant value in having a pure Python implementation. As the reviewer suggests "Python is at the moment probably the most widely used computer programming language by scientists". Having Python-Microscope in a language that the end user can code, invites them to look into the “box” and eases the process for these, possible casual, Python users to contribute with fixes and support for new devices.

Expected audience:

This manuscript will be of interest to those scientists who build/assemble their own microscope systems and write software code to control their operation.

Field of expertise:

I think a lot about microscope control software and how it can help scientists do their experiments.

Reviewer #2 (Significance (Required)):

see above.

Reviewer #3 (Evidence, reproducibility and clarity (Required)):

Pinto et al present a new python based software to control microscopes.
Overall the work is very interesting and will help microscopists to accelerate their development by providing new tool to integrate the different hardwares.

A few aspects commented below need to be clarified to help potential future users to integrate the software for the correct microscopes/hardware.

© 2021. Published by The Company of Biologists under the terms of the Creative Commons Attribution License (https://creativecommons.org/licenses/by/4.0/).

In general the software is mostly targeted to developers that want to build microscopes, as they mention in the discussion. Some positive features are (1) the ability to have experiments as scripts, (2) the software triggering, (3) the device-server structure, and (4) the ability to have virtual devices to try out the code and the testing I see in the github page. I think it's robust especially and mostly for the device-layer of the software. It's also positive that one can install it in python and import it in your programs, so it can be incorporated into other software fairly easy.

1) I miss more information regarding the latency of the device-server and software triggering, how fast can it be? How much delay would you have between computers/devices? For example, could we have the devices sincronized at the microsecond range? I think this is super important so that the reader knows if it's worth using a software triggering approach with Python-Microscope or they should buy a DAQ instead.

We generally expect high performance hardware to require hardware triggering, software triggers are very unlikely to be performant, or reliable enough to achieve ms, yet alone, μs timing accuracy and reproducibility. Software triggering is implemented as a basic approach to allow simple low speed hardware control, such as basic image snapping. Our systems all utilise external timing devices to provide digital triggering and, in some cases, analogue voltage control. This is becoming increasingly easy with high performance microprocessors such as the ardiuno or higher spec solutions such as National Instruments DAQ boards. We are currently investigating the recently released Raspberry Pi Pico boards, which provide very high performance digital triggering at very low cost (~£4). We are passionately promoting open source, low cost solutions, so requiring a NI DAQ board and LabView licenses goes against the spirit of this project.

1b) It's good though that they don't want to limit themselves to software triggering but also mention hardware triggering, but it's important to better explain where are the limitations.

This is a significant issue but we feel it is beyond the scope of this paper. We utilise microscope as a low level interface to hardware for our systems. The hardware control software has no internal knowledge of device connectivity eg which filter wheel might be in front of what camera, so any integrated control, such as synchronising light sources and cameras is beyond the scope of this package. We use the cockpit package as a GUI and to provide this higher level control integration. We then utilise hardware timing devices interfaced to cockpit to run experiments. We feel that this is a relatively cheap and approachable solution while allowing high performance from even complex systems.

1c) Needs info adding to the text, but in general python-microscope doesn't concern itself with this, just allows setting of trigger types and you are then responsible for triggering.

As suggested by the reviewer, Python-Microscope does not generally concern itself with triggering. It allows setting of trigger types in a consistent manner, and on relevant devices can initiate a software trigger event. The end of the section "Fast and furious" now reads:

"The microscope interface was designed with the concept of triggers that activate the individual devices and software triggers are handled as simply another trigger type. This approach provides an interface that supports software triggers but is easily upgraded to hardware triggers. The source of such hardware triggers can be other devices --- typically a camera --- or a dedicated triggering device. The recommended procedure is to prepare an experiment template that is then loaded on a dedicated timing device which triggers all other devices, as described in Carlton 2010. The existence of fast and cheap microprocessors and single board computers mean providing a dedicated hardware timing to sequence and synchronise a number of devices is relatively easy and extremely cost effective. We would recommend systems are designed around using an external device to provide hardware triggers to devices. This provides reliable timing and much more flexible sequencing than directly connecting outputs from one device to trigger inputs for another."

© 2021. Published by The Company of Biologists under the terms of the Creative Commons Attribution License (https://creativecommons.org/licenses/by/4.0/).

1d) I also miss information about the triggering, do the software offer a platform that can synchronize devices, or that's more left to the developer to do? They say they can generalize to arbitrarily complex devices so therefore I think it needs to be specified how. Same with the server feature, how fast is that link?

The software triggering depends very much upon the individual devices and delays such as context switching within the OS. We offer no solution to synchronise devices. Our claim to generalise to arbitrarily complex systems is based on the fact that you can trivially run devices on different computers to allow horizontal scaling. If you wish to have 25 cameras, simply run them on different computers, then none will be speed limited by computational resources. Synchronisation can be achieved by an external hardware timing device as described above.

The server link is passed over standard ethernet, likely now 1GB/s, however data packets must be serialised before transmission and deserialised on receipt by Python, as well as standard network overhead and latency. We have only seen network limitations on image transfer from cameras to remote server computers. This has not been a significant issue as the cameras drivers typically have memory buffers, which can be enlarged to cope with backlogs, as well as the Python-Microscope image transmission processes acting on a FIFO memory queue. Possibly long experiments utilising fast, high pixel count cameras could saturate these buffers, but such a specialised application could use specialised solutions such as multi-path networking or a computer with a very large amount of RAM for temporary buffering.

2a) Some critical comments are that, first of all there are not so many drivers yet available (for example Hamamatsu camera).

The reviewer is correct, device support is critical. There are two components to this, a) the resources to implement new devices, and b) the physical hardware to enable testing and debugging of these devices. We have focused on the hardware that we own and use but hardware support is expanding. As described in our reply to reviewer #1, we hope that a community of experienced hardware and software developers will evolve and help support new devices. We have instructions on how to support new hardware devices and are happy to help interested parties. We also plan to apply for continuing funding to enable us to further develop Python-Microscope, especially to expand its range of supported hardware,

The well defined interface with the abstract base class in Python enforces what is required for a minimal implementation of a specific device type. Most devices are relatively easily supported by reference to existing devices of the same type. For instance, a stage is likely to be communicated to by serial over USB, taking simple text commands and returning easy to interpret responses. Adding a new device simply involves defining what commands to send and how to deal with the replies from the hardware. With a suitable manual this can typically be done with a few hours of programming and testing.

2b) I guess this paper is also to show proof of concept and then upon interest they will include more devices, but in that case it should be more documented how one can contribute to the project and generate new drivers. For example, if we want to try it tomorrow in our setups, and we have a specific device such as an Hamamatsu camera, What should we do? Should we contact the authors, write an issue in the github page or write the driver ourself?

We have added the following paragraph on contributing to the project at the end of discussion section of the paper:

Microscope is a free and open source project currently being used in several labs with an open development approach. Our aim is that the microscope development community will find it a useful tool and engage in this development to increase its general usefulness. With that aim in mind, we perform our development conversations and user support in the open as github issues and the project is an image.sc community partner. In particular, expanding the number of devices supported by Microscope would be extremely beneficial. However, adding support for a device requires physical access to the device and the current list of supported devices echoes the devices we and our collaborators have access to. This is a chicken and egg problem.

© 2021. Published by The Company of Biologists under the terms of the Creative Commons Attribution License (https://creativecommons.org/licenses/by/4.0/).

Python-Microscope needs broad device support to be widely adopted by the community but it needs contributions from the community to support those devices. We believe that, Microscope currently provides enough devices and infrastructure to support adoption by more developers. There are contribution guidelines within the ``Get Involved'' section of the documentation, available online at https://www.python-microscope.org/doc/get-involved.

3) Second, the graphical interface is maybe good enough for developers and builders but in order to have a solid microscope that biologists are going to use it needs a bit more work in that direction.

The GUI in microscope is extremely basic and designed for quick testing. For a microscope system aimed at biological users we would recommend using Microscope-Cockpit, our paper is now referenced and a supplemental figure shows an example of its interface, or implementing an alternative more specialised GUI. We have released Python-Microscope as a separate package to separate low level hardware control from a GUI front end, enable relatively easy automated control of microscope systems directly from Python, or allow others to create GUI base interfaces without having to deal with interfacing to specific hardware.

Reviewer #3 (Significance (Required)):

Microscope control software, especially is open source, can help the rapid integration of new hardware and accelerate overall microscopy development.

I see this paper as an important starting point platform for future more user friendly Python-microscope controlling software.

Original submission

First decision letter

MS ID#: JOCES/2021/258955

MS TITLE: Python-Microscope: A new open source Python library for the control of microscopes

AUTHORS: David Miguel Susano Pinto, Mick A Phillips, Nicholas Hall, Julio Mateos-Langerak, Danail Stoychev, Tiago Susano Pinto, Martin J Booth, Ilan Davis, and Ian M Dobbie
ARTICLE TYPE: Research Article

We have now reached a decision on the above manuscript.

To see the reviewers' reports and a copy of this decision letter, please go to: https://submit-jcs.biologists.org and click on the 'Manuscripts with Decisions' queue in the Author Area. (Corresponding author only has access to reviews.)

As you will see, the reviewers gave favourable reports but reviewer #2 raised some critical points that will require amendments to your manuscript. I hope that you will be able to carry these out because I would like to be able to accept your paper, depending on further comments from reviewers.

We are aware that you may be experiencing disruption to the normal running of your lab that makes experimental revisions challenging. If it would be helpful, we encourage you to contact us to discuss your revision in greater detail. Please send us a point-by-point response indicating where you are able to address concerns raised (either experimentally or by changes to the text) and where you will not be able to do so within the normal timeframe of a revision. We will then

© 2021. Published by The Company of Biologists under the terms of the Creative Commons Attribution License (https://creativecommons.org/licenses/by/4.0/).

provide further guidance. Please also note that we are happy to extend revision timeframes as necessary.

Please ensure that you clearly highlight all changes made in the revised manuscript. Please avoid using 'Tracked changes' in Word files as these are lost in PDF conversion.

I should be grateful if you would also provide a point-by-point response detailing how you have dealt with the points raised by the reviewers in the 'Response to Reviewers' box. Please attend to all of the reviewers' comments. If you do not agree with any of their criticisms or suggestions please explain clearly why this is so.

Reviewer 1

Advance summary and potential significance to field

Note: I am reviewer #1 in the “Response to Reviewers” pdf

Summary and potential significance:

As stated in my first review of the manuscript, Pinto et al. report about a new open sources Python library that allows scientists to write microscope acquisition code faster. It includes classes for various devices that are already implemented and do not have to be written from scratch, supports hardware and software triggers, provides several developer-friendly features, etc.

Although microscopy builders have already used Python and other Open Science software platforms to write acquisition software for quite some time, the written code was often too specific, too inflexible, not user- and developer-friendly, and/or not well-documented, making the adoption and dissemination of such code challenging.

Therefore, the field of microscopy is missing the kind of library the authors developed.

Such a library will not only help to write acquisition code faster, but it will also (1) support the democratization of microscopy (scientists might consider building their own scopes (because it seems more feasible) than buying experience instrumentation) and (2) help with the reproducibility of imaging experiments (Python is by nature an open software platform which would allow the sharing of code without violating company licenses, etc.).

Therefore, I consider the paper significant for the microscopy and imaging field.

Reviewer's signature: Ulrike Boehm

Comments for the author

Note: I am reviewer #1 in the “Response to Reviewers” pdf
I would like to thank the authors that most of my previous comments have been addressed and integrated into the manuscript which improved its overall quality.

But the authors might want to tackle two minor points:
(1) Renaming of the "Fast and furious" section into, e.g., "Hardware and software trigger integration" to be more specific.

(2) The authors renamed "Microscope" into "Python-Microscope" as requested but have not been consistent in the renaming. Therefore, the entire document should be revised accordingly. E.g., the last paragraph of the discussion:
"Microscope is a free and open-source project..."
With these two minor changes, I consider the current manuscript suitable for publication.
Reviewer's signature: Ulrike Boehm

© 2021. Published by The Company of Biologists under the terms of the Creative Commons Attribution License (https://creativecommons.org/licenses/by/4.0/).

Reviewer 2

Advance summary and potential significance to field

This manuscript describes “Python-Microscope” an open source software library for control of microscope hardware. The distinguishing feature is that this library is written in Python, rather than the C++ language used in the hardware control layer of the more established software project “Micro-Manager”. The manuscript is very well written, and the software seems well designed, with a few interesting projects already making use of it. This library not only “competes” with Micro-Manager, but also with many other “home-grown” solutions. As the authors themselves state, the impact will eventually depend on wide spread adoption of the library, which is also needed to increase the number of supported devices, a “chicken and egg problem”.

Even though I support publishing this manuscript, I doubt that the scientific community is helped by having multiple software frameworks for microscope control. The resources to create and maintain support for a large number of devices is enormous, can not be accomplished by academic funding alone, and needs industry support. If we, as the scientific community, ask industry to support multiple interfaces (and it is difficult to imagine that we will stop at two, see for instance another microscope interface written in Python: https://github.com/openjournals/joss-papers/blob/joss.03241/joss.03241/10.21105.joss.03241.pdf), then it will be unsurprising if industry were to stop such efforts altogether. I may be idealistic in my desire to collaborate rather than compete, but especially in this case the similarities in the device interface design are much greater than the differences, and the cost of duplication is high. I also believe that the main difference (using Python as the programming language rather than C/C++) in the long term will turn out to be a handicap rather than an advantage; whereas C/C++ interfaces can be used from any programming language, Python code can be used from Python, but not other environments that very well may become more popular in the future (such as Julia). Efforts are under way to update and modernize the Micro-Manager interface (https://github.com/micro-manager/futureMMCore), and plans exist to make it possible to write Micro-Manager device adapters in Python. I hope that the relatively small community of software developers working on microscope control can collaborate rather than each go their own way.

Nico Stuurman

Comments for the author

- Does Python-Microscope contain some kind of abstraction for a hardware timing device, or are these completely left by themselves?
- Is there a speed penalty for the device-server architecture (when run on a single computer)? What are the highest data rates possible on this architecture?

Reviewer 3

Advance summary and potential significance to field

An open source microscope control software able to make use of a large library of devices is needed and will accelerate the development of new technology.

Comments for the author

The authors provided valuable explanation to my previous criticism.
I therefore support the work for publication in the current status.

© 2021. Published by The Company of Biologists under the terms of the Creative Commons Attribution License (https://creativecommons.org/licenses/by/4.0/).

First revision

Author response to reviewers' comments

Response to reviewers
We would like to thank the reviewers for their constructive and helpful comments. Our responses to reviewers specific comments are included below. Reviewers comments are in black and our responses are in red, along with specific changes to the text.

Reviwer #1 - Ulrike Boehm

(1) Renaming of the "Fast and furious" section into, e.g., "Hardware and software trigger integration" to be more specific.

We have made the suggested change.

(2) The authors renamed "Microscope" into "Python-Microscope" as requested but have not been consistent in the renaming. Therefore, the entire document should be revised accordingly. E.g., the last paragraph of the discussion: "Microscope is a free and open-source project..."

We have checked and ensured that “Microscope” has been changed to “Python-Microscope” in all the relevant places.

Reviewer #2 - Nico Stuurman

In response to this reviewer’s general point about supporting hardware on multiple software approaches, we agree and have added the following text to the discussion:

"In an effort to minimise duplication and increase community cooperation we are keen to foster links and collaborations with other Python based, or Python compatible microscope control packages such as PYME and μManager. We will endeavour to make bindings, when possible, that will enable interoperability of Python-Microscope with other open community-based Python software controlling microscope hardware."

(1) Does Python-Microscope contain some kind of abstraction for a hardware timing device, or are these completely left by themselves?

No Python-Microscope does not contain any hardware timing abstraction. We have considered this but decided that the instrument configuration information is not present in microscope and should be at a higher level of abstraction, so the hardware timing should also be. We have such an abstraction associated with our GUI control software, Microscope-Cockpit, sitting at a higher level and using Python-Microscope. A parallel manuscript describing Microscope-Cockpit is currently under review at Wellcome Open Research (https://wellcomeopenresearch.org/articles/6-76). The timing abstraction from Microscope-Cockpit has 3 concrete implementations on 1) a dedicated DSP board 2) National Instruments FPGA board and 3) Red Pitaya, a Xilinx manufactured single board computer. All 3 provide hardware timed output for both digital trigger and analogue signals.

(2) Is there a speed penalty for the device-server architecture (when run on a single computer)? What are the highest data rates possible on this architecture?

This is actually a complex question and doesn't come with a simple answer as it depends on both hardware and operating system implementations. We performed some benchmark tests with a simulated camera returning black images (all pixel values 0) at 512x512 pixels and 8 bits/pixel. The script times the generation and then passing of 1000 images, and averages to get time per frame. Below I attach some benchmarks from systems to compare.

© 2021. Published by The Company of Biologists under the terms of the Creative Commons Attribution License (https://creativecommons.org/licenses/by/4.0/).

Times are in ms/frame.

OS	local access time	remote access time
Windows 10	31.4	31.1
macOS	2.54	2.04
Linux	2.45	1.94
Linux (raspberryPi)	8.04	20.3

The Windows, Linux and macOS systems end up with negative additional overhead, presumably because the Python GIL is being bypassed by two separate Python processes spread across multiple cores on fast, memory rich multi-core computers. However, note that the Linux and macOS version of the identical python code run more than 10 times faster than the Windows version. On Linux and macOS local access allows about 400 frames/sec while remote is about 500 frames/s. We also include a Linux example is on a relatively low powered Raspberry Pi system. This shows that resource limits can change the balance between the two image access modes, possibly because the limited memory of the Raspberry Pi.

Reviewer #3

No queries

Second decision letter

MS ID#: JOCES/2021/258955

MS TITLE: Python-Microscope: A new open source Python library for the control of microscopes

AUTHORS: David Miguel Susano Pinto, Mick A Phillips, Nicholas nicholas.hall@strath.ac.uk Hall, Julio Mateos-Langerak, Danail Stoychev, Tiago Susano Pinto, Martin J Booth, Ilan Davis, and Ian M Dobbie
ARTICLE TYPE: Research Article

Thank you for sending your manuscript to Journal of Cell Science through Review Commons. I am happy to tell you that your manuscript has been accepted for publication in Journal of Cell Science, pending standard ethics checks.

© 2021. Published by The Company of Biologists under the terms of the Creative Commons Attribution License (https://creativecommons.org/licenses/by/4.0/).
